# High-frequency neuronavigated rTMS effect on clinical symptoms and cognitive dysfunction: a pilot double-blind, randomized controlled study in Veterans with schizophrenia

**DOI:** 10.1038/s41398-020-0745-6

**Published:** 2020-02-25

**Authors:** Heng Yong Guan, Jian Min Zhao, Ke Qiang Wang, Xiu Ru Su, Yan Fen Pan, Jin Ming Guo, Long Jiang, Yu Hong Wang, Hong Yu Liu, Shi Guang Sun, Hao Ran Wu, Yan Ping Ren, Han Song Geng, Xiao Wen Liu, Hui Jing Yu, Bao Chun Wei, Xi Po Li, Hanjing Emily Wu, Shu Ping Tan, Mei Hong Xiu, Xiang Yang Zhang

**Affiliations:** 1 Hebei Province Rong-Jun Hospital, Baoding, China; 2 The National Clinical Research Center for Mental Disorders & Beijing Key Laboratory of Mental Disorders, Beijing Anding Hospital, Capital Medical University, Beijing, China; 3 Department of Psychiatry and Behavioral Sciences, Baylor College of Medicine, Houston, TX USA; 4 Peking University Huilongguan Clinical Medical School, Beijing Huilongguan Hospital, Beijing, China; 5 Department of Psychology, University of Chinese Academy of Sciences, Beijing, China; 6 CAS Key Laboratory of Mental Health, Institute of Psychology, Chinese Academy of Sciences, Beijing, China

**Keywords:** Schizophrenia, Scientific community

## Abstract

Cognitive impairment is a central aspect of schizophrenia (SCZ) that occurs at the onset of the disease and is related to poor social function and outcome in patients with SCZ. Recent literatures have revealed repetitive transcranial magnetic stimulation (rTMS) to be one of the efficient medical interventions for cognitive impairments. However, no study has been conducted to investigate the treatment effectiveness of 20 Hz rTMS with neuronavigation system administered to the left dorsolateral prefrontal cortex (DLPFC) in patients with schizophrenia. In this randomized, double-blind and sham-controlled study, 56 patients were enrolled in 20 Hz rTMS (*n* = 28) or sham stimulation (*n* = 28) over left DLPFC for 8 weeks. The Repeatable Battery for the Assessment of Neuropsychological Status (RBANS) was performed to measure the cognitive function at baseline and after 8 weeks of rTMS treatment. The positive and negative syndrome scales (PANSS) was performed to assess the clinical symptoms at baseline, after 2-week treatment, 4-week treatment, 6-week treatment, and 8-week treatment. Totally, 15 subjects (seven in active group and eight in sham group) dropped out during the trial and the main findings were from completed 41 patients. At 2 weeks, 4 weeks, and 6 weeks, there were no significant differences in PANSS total score and subscores between the sham and treatment groups. At 8 weeks, the 20 Hz rTMS significantly increased the immediate memory score compared with the sham. Furthermore, the improvement in the immediate memory score was correlated with the decrease in the excitement factor score of the patients with SCZ. Our results suggest that 20 Hz rTMS appears to be an effective treatment for improving the cognitive performance and reducing the clinical symptoms of patients with SCZ.

## Introduction

Schizophrenia (SCZ) is a chronic mental illness that affects about 1% of the whole population. Many antipsychotic medications have a good effect on the vast majority of positive symptoms of patients in any phase of illness, but limited effect on negative symptoms or cognitive dysfunction^1^. Negative symptoms in SCZ are characterized by low motivation, lack of speech, and little interest in social behavior. Particularly, patients with SCZ display a general impairment in cognitive function including working memory, executive function, selective attention, immediate memory, and delayed memory^2–4^. Both cognitive impairment and negative symptoms strongly and negatively impact daily functioning and determine social functioning throughout the lives of the patients with SCZ^5^. However, the current antipsychotic treatments have almost little or no effect on these symptoms, in particular cognitive impairment^6^.

Repetitive transcranial magnetic stimulation (rTMS) is recognized as an augmentation therapy for some symptoms of SCZ, who failed multiple pharmacologic interventions. rTMS has been shown to be effective in numerous severe neuropsychiatric diseases such as bipolar disorder, depression, and anxiety^7^. In SCZ, several double-blind, randomized control trials have been employed to investigate the effect of high-frequency rTMS treatment to stimulate the excitability of left dorsolateral prefrontal cortex (DLPFC) on negative symptoms of SCZ^8–10^. For example, a recent meta-analysis including 827 participants reported that the rTMS treatment target on the frontal cortex had a mean weighted effect size (ES) of 0.64^11^. In addition, the study also suggested that rTMS is effective in treating negative psychopathological symptoms in SCZ^11^. However, an earlier meta-analysis revealed no statistically significant difference in reduction of negative symptom scores between active rTMS group and sham group^12^. Collectively, all these results showed that further rTMS studies for systematically assessing psychiatric symptoms in certain subtypes of SCZ are warranted due to significant differences across numerous participants recruited in the different studies.

Conversely, only a few of studies reported the effects of rTMS treatment on cognitive dysfunction in SCZ^13–15^. For example, an open-label study showed that rTMS targeting to the left DLPFC (10 Hz) and left temporoparietal cortex (1 Hz) significantly improved auditory verbal memory^16^. Wolwer et al. indicated that 10 Hz rTMS stimulation to the left DLPFC significantly improved facial emotion recognition in SCZ^17^. In particular, rTMS treatment bilaterally sequentially to left and right DLPFC was shown to significantly improve the accuracy of 3-back test compared with sham^15^. However, the recent double-blind, sham-controlled trials of rTMS in patients with SCZ showed that 10 Hz rTMS targeting to the left DLPFC for 3 weeks was not superior to sham rTMS in the improvement of numerous cognitive domains^18^. Moreover, a most recent meta-analysis demonstrated that prefrontal rTMS exerted beneficial effects on attention and executive deficits in certain depressive patients but not in the patients with SCZ^19^. It should be noted that the treatment protocols regarding rTMS efficiency are not consistent. This may explain the dramatically significant difference between studies reported in prior meta-analyses. For example, technical differences in rTMS treatment protocols including the localization of treatment (left versus right versus bilateral), duration of treatment, stimulation frequency, accuracy of targeting, and patient characteristics may lead to different treatment effect of rTMS^11,20^. Previous studies have focused primarily on stimulation frequencies of 10 Hz rTMS for 4 weeks, but found to be less effective in SCZ, especially for cognitive dysfunction. It is still unclear whether more repetitions or increased number of total pulses yield better results. Longer course of treatment and higher frequency of stimulation indicate more doses and more pulses of rTMS on DLPFC of the patients.

Due to the anatomic variation between patients, rTMS efficacy may be improved through a precise target^21^. For instance, most studies have targeted to the left DLPFC due to its central role in working memory and in executive function^22^. However, DLPFC is a comparatively large cortical area, which is neither accurate nor easy to target at this anatomical location. To date, most rTMS studies target based on the approximated “5–7-cm methods”, which have proposed to target 5–7-cm anterior to the primary motor cortex^23–25^. However, literatures have revealed that the vast majority of treatment targets may not be accurate in the DLPFC^26,27^. Thus, further studies are needed to optimize rTMS targeting techniques to improve treatment efficacy. Neuronavigation system is an indispensable instrument for accurate planning, targeting, and monitoring in brain stimulation studies. Using rTMS, the system co-registers the patient’s head to a standardized brain^28^. Under the guidance of the navigation system, the technicians can exactly observe the relative location of the magnetic coil of rTMS to the individual’s brain. The entire stimulation process is completely visualized, and the detailed parameters of each stimulus can be saved as images. Therefore, the neuronavigation system allows for precise, optimal, and reproducible targeting and stimulation of the DLPFC sites.

Several recent literatures have shown some efficacious results by combining the high-frequency rTMS with neuronavigated target on auditory verbal hallucinations in SCZ, relative to sham stimulation^21^. This study was the first one to assess the treatment efficiency of 20 Hz rTMS for the clinical symptoms and cognitive dysfunction in patients with SCZ. We proposed that: (1) the combination of neuronavigation, high-frequency (e.g., 20 Hz) stimulation and longer treatment duration (8 weeks) may be an optimal strategy for improving cognitive function and reducing negative symptoms in patients with SCZ; (2) the rTMS-induced cognitive improvement may be associated with changes in certain symptoms that share a similar, yet separable etiology to the cognitive impairment.

## Methods

### Participants

All participants were recruited in HeBei Province Rong-Jun Hospital in Northern China. The clinical trial protocol was approved by the Institutional Review Board of HeBei Province Rong-Jun Hospital. After receiving the full explanation on the purpose of the current study, every participant signed written informed consent form prior to recruitment. Fifty-six inpatients of ages 20–60 were recruited and diagnosed with SCZ by the Structured Clinical Interview for DSM-IV, without any patients with schizoaffective disorder. The patients also met the following inclusion criteria: (1) male; (2) right-handed; (3) Han Chinese; and (4) positive score on positive and negative syndrome scale (PANSS) < 24 and PANSS negative symptom score ≥ 20; and (5) ≧5-year duration of illness.

At baseline, we recorded a complete medical history of all patients. Recruited participants had no recent life stressors or clinically significant emotional abnormalities for at least 1 month prior to participation in the current study, which were obtained through the questions we asked them verbally. Subjects with physical diseases or cerebral pathologies including multiple sclerosis seizure, dementia, epilepsy, aneurysm, Huntington’s disease, brain tumor, stroke, Parkinson’s disease, severe headache for unknown reasons, and cardiovascular diseases were excluded. Also, those who received electroconvulsive therapy or rTMS within the past 6 months were excluded. Past history of autoimmune diseases, allergies, hypertension, lung disease, diabetes, cerebrovascular disease, family history of epilepsy, pregnancy or breast feeding, education level <5 years by subject report, receiving or planning to start the psychotherapy during the rTMS treatment, or those who had received psychotherapy in the last 6 months before the current study were also excluded.

### Procedure

This study was a double-blinded and randomized controlled pilot trial. Of the 56 participants, 28 were in the active rTMS group (20 Hz) and 28 in the sham stimulation group. Briefly, subjects were assigned to receive 40 treatments of either 20 Hz or sham rTMS over the duration of 8 weeks.

After the recruitment, all participants were randomly and separately assigned to real (20 Hz) or sham rTMS condition. An independent, third party assorted participants into either the real or sham groups through computer generated randomization numbers that were compiled through simple randomization. The clinical staff and patients were blind to the assignment, except for one clinical technician, who provided the real rTMS or sham treatment according to the randomization numbers. Clinical assessment was performed at baseline and weeks 2, 4, 6, and 8. Cognitive performance was assessed at baseline and week 8. Raters who were blind to treatment status assessed clinical symptoms and cognitive function in the study.

### Neuronavigated rTMS treatment

For the exact stimulation locations of the left DLPFC, neuronavigation system (Polaris Vicra position sensor, BrainSight, Magstim, UK) was used for accurate planning, targeting, and monitoring. Brain images for each patient were collected with a 0.3 T magnetic resonance imaging (MRI) (BrivoMR235). The image data were imported to the neuronavigation system and then the treatment positions were accurately located accordingly. All processes were performed by a clinical technician.

In active group, 20 Hz stimulus on left DLPFC occurred at a power of 110% of motor threshold for 3-s intervals with 27-s inter-train interval. Forty sessions of treatment were administered five times a week (Monday to Friday) for 8 weeks using a MagStim Rapid stimulator (total stimuli = 64,000) (MagStim Company Ltd). In the sham group, all treatment procedures were the same as those in the 20 Hz group except for a false coil (P/N: 3950-00, Magstim Co.), which is different from the MagVenture CoolB65 Active/Placebo coil (P/N: 9016E0501; MagVenture A/S) in the intensity and distribution of the E-field in the subject’s head^29^. The false coil was placed in the same position as the active treatment. The sham treatment produced the same vibration as the true stimulus but no magnetic field and thus no therapeutic effect. Both real and sham administrations are identical in appearance and in sound.

All patients were on a stable dose of antipsychotic medication for at least 6 months before the study enrollment, which means that the doses of antipsychotics remained fixed for at least 6 months. Moreover, antipsychotics and all other medications remained fixed throughout the double-blind period. The medications that patients had been taking were clozapine (16 in rTMS versus 20 in sham groups), risperidone (five in rTMS versus seven in sham groups), olanzapine (three versus zero), chlorpromazine (two versus one), ziprasidone (one versus zero), and sulpiride (one versus zero). Six patients were taking antidepressant medications including escitalopram oxalate, sertraline, and paroxetine. There were no any differences in the antipsychotics (type and dose) between two groups. In addition, chlorpromazine equivalent for every participant was calculated as described in other literatures in detail^30^.

### Clinical symptom and neuropsychological assessments

The psychopathology of patients was assessed by three clinically trained staff using PANSS after a training session to ensure consistency and reliability of ratings. A PANSS five-factor model of “positive”, “negative”, “cognitive”, “emotional/depressed”, and “excited” was used in the current study^31^. The components are: positive component (P1 + P3 + P5 + G9); negative component (N1 + N2 + N3 + N4 + N6 + G7); cognitive component (P2 + N5 + G11); emotional/depressed component (G2 + G3 + G6); and excited component (P4 + P7 + G8 + G14).

The Repeatable Battery for the Assessment of Neuropsychological Status (RBANS) was used to assess the cognitive function, which contains five subtests of immediate and delayed memory, visuospatial/construction, attention, and language. A translated and adapted Chinese version of RBANS had been evaluated for reliability and clinical validity in our group^32^. Three psychologists assessed the cognitive impairment of the patients using the RBANS.

### Data analysis

The data were described using the means ± standard deviations and *p* values ≤ 0.05 were considered to be statistically significant. The demographic characteristics, clinical symptoms, and neuropsychological test scores were analyzed between two groups at baseline using analysis of variance (ANOVA) or the chi-square test. The intention-to-treat analysis was carried out and missing outcome data were entered as the principle of last observation carrying forward.

To investigate the effect of 20 Hz rTMS on cognitive performance and clinical symptoms in patients, the main strategy involved repeated-measures multivariate analyses of variance in the longitudinal study. The primary outcome was cognitive function measured by RBANS and clinical symptoms by PANSS. For the dependent variables, five time points (weeks 0, 2, 4, 6, and 8) were used as the repeated measures within-effect, and group (20 Hz versus sham) was used as the between-effect. If the group × time interaction was significant, then the group differences at weeks 4, 6, and 8 were respectively analyzed by analysis of covariance (ANCOVA) and the baseline scores were covariates. If the above interaction effect was not significant, further statistical testing was not required.

The second aim was to determine whether improvement of cognitive function correlated with reduction of PANSS scores. Therefore, the changes in the RBANS scores and PANSS scores in the two groups (real/sham) before and after the 8-week treatment were calculated for each patient. Correlations between cognitive improvement and reductions in PANSS scores were analyzed and the Bonferroni corrections were applied when significant. Finally, we used multiple linear regressions to investigate the potential response predictors associated with changes in the cognitive scores and clinical symptoms.

## Results

### Demographic and basic descriptive data

At baseline, as shown in Tables 1 and 2, no difference was found between rTMS and sham groups in the demographic characters, clinical variables including the PANSS scores, as well as the RBANS total and subscores. Interestingly, RBANS total score significantly negatively correlated with positive subscale score (*r* = −0.284, df = 56, *p* = 0.04), and negative subscale score (*r* = −0.441, df = 56, *p* = 0.001).

**Table 1:** Demographic data in active and sham groups.

	20 Hz (*n* = 28)	Sham (*n* = 28)	*X*^2^ or *F* (*p* value)	20 Hz (*n* = 21)	Sham (*n* = 20)	*X*^2^ or *F* (*p* value)
Age (years)	51.9 ± 10.1	56.0 ± 7.3	2.9 (0.09)	55.5 ± 7.3	49.3 ± 10.2	5.1 (0.03)
Education (years)	7.9 ± 2.6	7.8 ± 1.8	0.01 (0.99)	7.9 ± 1.8	8.2 ± 2.3	0.1 (0.71)
Age of onset (years)	20.1 ± 2.4	21.1 ± 1.7	0.2 (0.86)	21.1 ± 1.8	20.7 ± 2.4	0.4 (0.55)
Duration of illness	31.3 ± 9.7	34.5 ± 6.9	2.4 (0.95)	34.3 ± 6.8	30.4 ± 0.5	2.1 (0.16)
Hospital time	6.3 ± 3.2	5.6 ± 3.1	0.4 (0.70)	5.3 ± 3.0	5.5 ± 2.6	0.04 (0.85)
Antipsychotics						
Clozapine	16	20		13	15	
Risperidone	5	7		3	4	
Olanzapine	3	0		3	0	
Chlorpromazine	2	1		2	1	
Sulpira	1	0		0	0	
Ziprasidone	1	0		0	0	
DAD (mg)	413.9 ± 226.5	424.5 ± 288.3	1.2 (0.31)	420.4 ± 221.3	403.5 ± 227.6	1.6 (0.23)
PANSS total score	72.3 ± 12.9	79.9 ± 16.9	2.3 (0.10)	75.1 ± 14.9	71.2 ± 13.0	0.8 (0.38)
P-subscore	11.3 ± 4.5	11.4 ± 3.6	0.3 (0.71)	10.7 ± 2.6	11.5 ± 4.8	0.5 (0.50)
N-subscore	28.7 ± 6.7	31.9 ± 8.4	2.4 (0.10)	29.4 ± 7.4	27.4 ± 6.3	0.9 (0.35)
G-subscore	32.3 ± 7.6	36.6 ± 8.7	2.4 (0.10)	34.9 ± 8.3	32.3 ± 7.7	1.2 (0.29)
RBANS total score	58.3 ± 12.1	61.5 ± 12.7	0.8 (0.37)	60.1 ± 12.5	64.1 ± 12.7	1.1 (0.31)
Immediate memory	52.0 ± 12.9	50.1 ± 10.5	0.2 (0.83)	51.8 ± 10.9	53.9 ± 13.9	0.3 (0.59)
Attention	70.8 ± 16.4	66.8 ± 13.5	0.5 (0.60)	67.6 ± 13.4	74.6 ± 14.8	2.5 (0.12)
Visuospatial/ constructional	72.2 ± 17.2	69.9 ± 15.5	0.2 (0.85)	72.9 ± 14.8	74.9 ± 17.7	0.2 (0.70)
Delayed memory	65.4 ± 20.1	64.2 ± 20.3	0.1 (0.96)	66.8 ± 20.1	68.8 ± 19.3	0.1 (0.75)
Language	79.3 ± 15.2	74.4 ± 15.8	0.7 (0.48)	75.8 ± 15.6	80.1 ± 15.3	0.8 (0.38)

*DAD* daily antipsychotic dose (mg) (chlorpromazine equivalent), *PANSS* Positive and Negative Syndrome Scale, *P* positive symptom, *N* negative symptom, *G* general psychopathology, *RBANS* repeatable battery for the assessment of neuropsychological status.

**Table 2:** PANSS total score and subscores at baseline, week 2, week 4, week 6, and week 8 in 20 Hz rTMS and sham groups.

	Baseline (*n* = 56)	Week 2 (*n* = 41)	Week 4 (*n* = 41)	Week 6 (*n* = 41)	Week 8 (*n* = 41)	Group *F* (*p* value)	Time *F* (*p* value)	Group × time *F* (*p* value)
PANSS total score						0.57 (0.57)	37.1 (0.00)	1.0 (0.39)
Sham	79.9 ± 16.9	70.5 ± 17.6	67.8 ± 20.3	67.3 ± 22.4	66.8 ± 22.7			
20 Hz	72.3 ± 12.9	67.2 ± 12.3	62.8 ± 13.7	60.6 ± 14.3	59.2 ± 14.4			
P-subscore						0.54 (0.58)	0.04 (0.83)	0.06 (0.94)
Sham	11.4 ± 3.6	10.4 ± 2.6	10.3 ± 2.6	10.1 ± 2.8	10.0 ± 2.8			
20 Hz	11.3 ± 4.5	11.4 ± 4.8	11.3 ± 4.6	10.9 ± 4.3	10.7 ± 4.3			
N-subscore						0.1 (0.55)	5.9 (0.00)	0.68 (0.58)
Sham	31.9 ± 8.4	26.1 ± 8.5	24.8 ± 9.3	24.5 ± 9.8	23.8 ± 10.0			
20 Hz	28.7 ± 6.7	25.3 ± 5.9	22.7 ± 6.2	21.5 ± 5.9	20.9 ± 5.9			
G-subscore						1.3 (0.28)	16.8 (0.00)	1.3 (0.28)
Sham	36.6 ± 8.7	34.0 ± 8.6	32.7 ± 9.9	32.7 ± 10.8	33.0 ± 10.9			
20 Hz	32.3 ± 7.6	30.6 ± 7.1	28.9 ± 7.2	28.3 ± 7.3	27.6 ± 7.5			
Postive factor						0.72 (0.49)	41.1 (0.00)	0.58 (0.65)
Sham	5.9 ± 2.5	5.1 ± 1.6	5.0 ± 1.4	5.2 ± 1.7	5.2 ± 1.7			
20 Hz	6.6 ± 3.4	6.6 ± 3.2	6.7 ± 3.1	6.6 ± 2.9	6.6 ± 3.0			
Negative factor						0.72 (0.49)	41.1 (0.00)	0.58 (0.65)
Sham	26.7 ± 6.9	21.9 ± 7.5	20.6 ± 8.3	20.0 ± 8.5	19.6 ± 8.7			
20 Hz	23.8 ± 6.0	20.8 ± 5.7	18.5 ± 5.7	17.5 ± 5.4	17.0 ± 5.5			
Cognitive factor						1.6 (0.20)	12.9 (0.00)	0.93 (0.44)
Sham	11.1 ± 3.8	9.8 ± 3.8	9.3 ± 4.2	9.6 ± 4.5	9.3 ± 4.8			
20 Hz	8.8 ± 2.5	8.2 ± 2.2	7.7 ± 2.5	7.4 ± 2.6	7.3 ± 2.7			
Excited factor						0.74 (0.48)	1.6 (0.28)	2.2 (0.074)
Sham	5.9 ± 2.5	5.5 ± 1.7	5.6 ± 1.7	5.6 ± 1.7	5.7 ± 1.8			
20 Hz	5.6 ± 2.2	5.3 ± 1.2	5.2 ± 1.7	4.8 ± 1.3	4.6 ± 1.1			
Depression factor						0.56 (0.58)	3.3 (0.02)	1.4 (0.20)
Sham	4.4 ± 1.9	4.3 ± 1.7	4.3 ± 1.7	4.2 ± 1.7	4.2 ± 1.6			
20 Hz	4.1 ± 1.9	4.3 ± 2.0	3.9 ± 1.9	4.1 ± 2.0	3.9 ± 2.0			

### rTMS treatment for cognitive performance

During the treatment, 15 subjects were discontinued because they withdrew their consent (three in active and four in sham rTMS groups) and MRI images could not completely be restored to 3D stereo images (four in active and four in sham rTMS groups). Thus, 41 patients completed the clinical trial, including 21 in active and 20 in sham rTMS groups.

In the primary outcome, repeated-measures ANCOVA on the immediate memory revealed a significant time effect (*F* = 7.6, df = 2, 74, *p* = 0.009), a nonsignificant group effect (*F* = 2.1, df = 1, 39, *p* = 0.15), and a significant interaction effect (*F* = 6.3, df = 2, 74, *p* = 0.017). ANCOVA showed that the immediate memory performance was higher in the rTMS group compared with the sham group at week 8 (*F* = 6.1, df = 1, 39, *p* = 0.018; ES = 0.36), after covarying for education, age, and dose of drug (chlorpromazine equivalents).

**Fig. 1:**
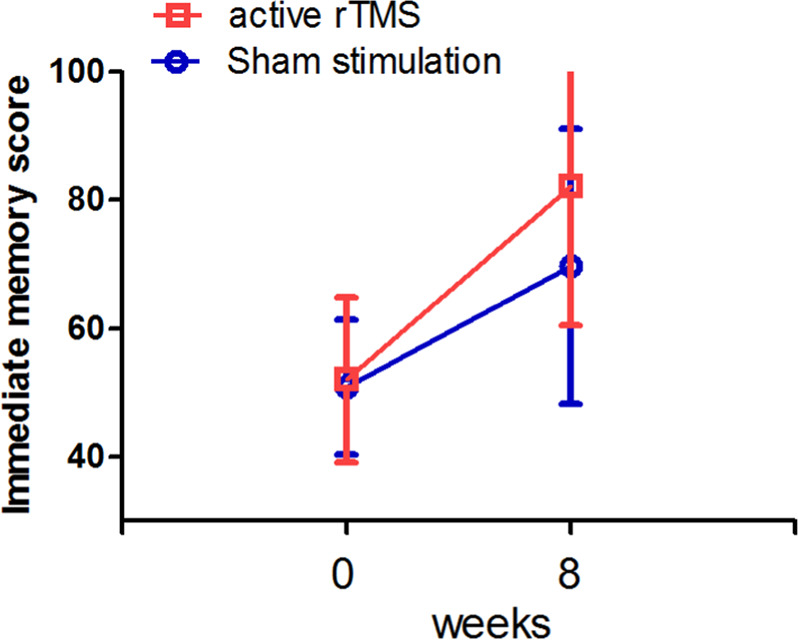
Comparison of immediate memory score between rTMS and sham groups before and after 8 weeks of treatment. rTMS treatment significantly increased the immediate memory score in the patients with schizophrenia, as compared with sham stimulation (*p* < 0.05).

Table 3 and Fig. 1 showed that the increase in immediate memory score from baseline to week 8 in the rTMS and sham groups was 28.2 ± 19.3 versus 16.0 ± 10.9, respectively (*F* = 6.3, df = 1, 42, *p* = 0.017). The mean difference in the change in the immediate memory index score from baseline to 8 weeks between the two groups was 12.2 ± 8.4 (ES = 0.78).

**Table 3:** Cognitive score and comparison at baseline and week 8 in 20 Hz and sham groups.

	Baseline (*n* = 56)		Week 8 (*n* = 41)		Group	Time	Group × time
	Sham	20 Hz	Sham	20 Hz	*F (p)*	*F (p)*	*F* (*p* value)
Immediate memory	50.9 ± 10.5	52.0 ± 12.9	69.7 ± 21.4	82.1 ± 21.6	3.3 (0.077)	52.1 (<0.001)	6.3 (0.01)
Attention	63.1 ± 15.4	70.5 ± 13.3	66.8 ± 13.5	70.8 ± 16.4	3.4 (0.07)	4.5 (0.04)	0.03 (0.96)
Visuospatial/constructional	69.9 ± 15.5	72.2 ± 17.2	78.5 ± 13.3	82.1 ± 18.6	0.43 0.52	10.1 0.003	0.08 (0.8)
Delayed memory	64.2 ± 20.3	65.4 ± 20.1	86.0 ± 18.5	90.7 ± 21.1	0.35 (0.56)	70.4 (<0.001)	0.34 (0.57)
Language	74.4 ± 15.8	79.3 ± 15.2	85.8 ± 17.0	84.6 ± 9.5	0.28 (0.60)	7.0 (0.012)	1.59 (0.22)
RBANS total score	58.3 ± 12.1	61.5 ± 12.7	71.3 ± 14.8	78.1 ± 16.2	2.5 (0.13)	81.0 (<0.001)	2.6 (0.11)

### rTMS treatment for psychopathological symptoms

**Fig. 2:**
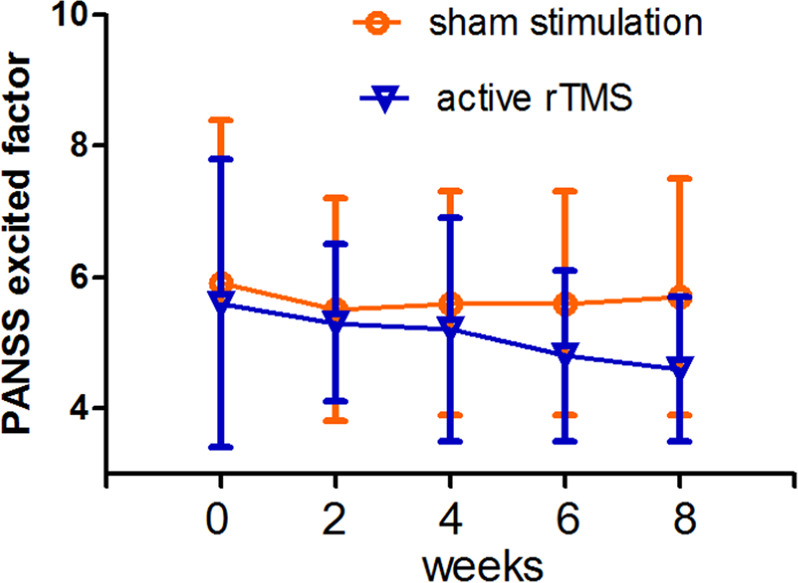
Comparison of PANSS excited factor between rTMS and sham groups before and after 8 weeks of treatment. rTMS treatment displayed a trendency to significance for PANSS excited factor in the patients with schizophrenia, compared with sham stimulation (*p* = 0.074).

In the secondary outcome, changes in the PANSS and their subscale scores are illustrated in Table 2 and Fig. 2. Repeated-measures ANOVA on PANSS and all its subscales showed a marginally significant interaction effect (group × time: *F* = 2.2, df = 1, 39, *p* = 0.074), nonsignificant group effect (*F* = 0.74, df = 1, 39, *p* = 0.48), and time effect (*F* = 1.6, df = 4, 39, *p* = 0.28). Further ANCOVA showed that the PANSS excited factor score was significantly more diminished in the rTMS group than in the sham group at week 6 (*F* = 3.5, df = 1, 39, *p* = 0.07; ES = 0.24) and at week 8 (*F* = 8.6, df = 1, 39, *p* = 0.006; ES = 0.39). The difference at week 8 remained significant after covarying for education, age, duration of illness, and dose of drug (chlorpromazine equivalents) (*F* = 7.4, df = 1, 37, *p* = 0.009).

No effect was found on the negative symptoms before and after treatment with rTMS after repeated-measures ANCOVA analysis.

### Correlation between the improvement of cognitive performance and change in psychopathological symptoms in 20 Hz rTMS group

Correlation analysis in 20 Hz rTMS group showed significant correlations between the increase in immediate memory from baseline to week 8 and the following parameters: the reduction of PANSS excited factor score (*r* = 0.42, df = 21, *p* = 0.05) and the positive subscore (*r* = 0.56, df = 21, *p* = 0.012). However, none of these significances survived Bonferroni correction (all *p* > 0.05).

Further analysis showed a trend toward significant association between increase in immediate memory index score and decrease of the positive subscore (beta = 7.5, *t* = 2.03, *p* = 0.07), after covarying for education, age, and dose of treatment.

## Discussion

The results of the current study showed that (1) rTMS produced an effective therapeutic benefit on immediate memory of patients with SCZ; (2) rTMS displayed an effect on PANSS excited factor, but not on negative symptoms; and (3) the improvement in immediate memory of patients was associated with the reduction in PANSS excited factor at 8th week.

In this pilot double-blinded, randomized controlled trial, real rTMS significantly increased immediate memory score, demonstrating a clinically meaningful improvement of immediate memory performance in patients with SCZ in the 20 Hz rTMS group. Recently, rTMS has been proposed as a new treatment option for cognitive dysfunction, which alters the neuronal activity in the applied area and related areas^6,18^. Most of the literatures showed that rTMS was effective on attention and executive functions in certain patients with depression^19^. But in SCZ, only two studies have reported the promising effect of rTMS on cognitive function as compared with sham control. One earlier study, by Mogg et al., reported a positive effect of 10 Hz rTMS applied to left DLPFC on the California verbal learning test^14^. Interestingly, another recent study revealed that bilateral 20 Hz rTMS treatment of DLPFC for 4 weeks improved working memory. Particularly, rTMS significantly improved the accuracy of target response to a level comparable to healthy subjects in the 3-back task in patients^15^. Our findings are similar to those reported in the above study in several points: first, the patients recruited in our study also had predominant negative symptoms; second, our results indicated that 20 Hz rTMS targeting on left DLPFC improved immediate memory performance in patients.

The exact mechanisms responsible for the effective treatment of rTMS for cognitive impairment are still unknown. Preclinical studies have shown that rTMS causes complex biochemical effects and neurotropic effects by reducing inflammation and oxidative stress^33,34^. For example, studies have revealed that rTMS increases the release of dopamine in certain brain pathways^35^, which is consistent with the hypoactive dopaminergic hypothesis in cognitive deficits of patients with SCZ^36^. Moreover, animal studies found that rTMS altered NMDA receptor concentration after a single stimulation, functionally as a potential agonist of the receptors^37^. Thus, the agonistic effect and upregulation of NMDA receptors by rTMS may improve the cognitive impairment. Second, a recent study showed that high-frequency rTMS may improve the impaired neuronal plasticity by activating BDNF pathway in ischemic rats^38^. Thus, high-frequency rTMS may improve cognitive deficits in SCZ patients by activation of neurotrophic factors, which play critical roles in neuroplasticity in the hippocampus related to learning and memory^39^. In sum, the potential mechanism of rTMS treatment on cognitive dysfunction may be due to those molecular effects to decorate the properties of neurons and increase the expression of neurotransmitters and their receptors, as well as activate neurotrophic factors^33^. However, these points are only our speculations since we did not measure the neurobiochemicals in the patients. The exact mechanisms underlying the effective treatment of 20 Hz rTMS for cognitive deficits in SCZ need further investigation.

Another finding in the present study was that 20 Hz rTMS over left DLPFC for 8 consecutive weeks displayed a trendency to significantly better therapeutic effect on excited factor of PANSS. Failure to improve the negative symptoms after treatment with rTMS is consistent with recent studies in other groups using rTMS targeting to left DLPFC for negative symptoms in patients with SCZ^12^. However, four other meta-analyses found significant effects of rTMS for negative symptoms of SCZ^7,8,40,41^. The discrepancies in the therapeutic effects of rTMS for clinical symptoms of the patients may be caused by duration of illness, different stages of disease progression (chronic versus acute), adjunctive antipsychotic medication, the type of outcome measures used, duration of rTMS treatment, as well as the techniques of rTMS, such as stimulus frequency, position and intensity of treatment, as well as the shape and dimension of coils.

Interestingly, we established a superior effect of active rTMS on the PANSS excited factor. Our further ANOVA analysis showed a significant difference in time × group interaction on poor impulse control (*p* < 0.05), indicating that the 20 Hz rTMS treatment has a positive effect on the PANSS excited factors compared with sham stimulation. A recent exploratory secondary analysis of the data from “rTMS for the Treatment of Negative Symptoms in Schizophrenia” (RESIS) trial reported that real rTMS significantly improved the PANSS excited factor, which was in accordance with our results^42^. However, further analysis in our study and the previous reanalysis study did not pass the Bonferroni corrections. This was due to the excited factor score consisting of four items of clinical symptoms that were already quite low prior to the treatment. Further longitudinal studies with larger samples in SCZ are warranted. Specifically, we found that the increase in the immediate memory score was correlated with the reduction in PANSS excited score at week 8, suggesting a close relationship between the improvements of cognitive deficits and clinical symptoms in patients with SCZ. At baseline, we found significant associations between the RBANS score and PANSS negative and positive scores, which provided further evidence for this point. Many studies support the notion that cognitive dysfunction is correlated with certain clinical symptoms in SCZ, indicating that they share common pathological mechanism^43^.

It is known that dopaminergic dysfunctions are associated with cognitive deficits and poor impulse control of SCZ^44^. Impaired ventral prefrontal cortex and DLPFC are interconnected with impulse controls^45^. Recent studies also showed that prefrontal dopaminergic dysfunction could lead to disturbances in striatal dopamine. Conversely, the striatal dopaminergic disturbances could cause the aggressive behaviors. Recent studies also supported the proposal that D_3_-preferred D_2_/D_3_ agonists are associated with impulsive control. As mentioned above, high-frequency rTMS could cause the release of dopamine in the mesostriatal brain pathways^46^, which may improve poor impulse control and cognitive deficits simultaneously. Indeed, we found a close relationship between the improvements of excited factor and cognitive deficits of SCZ with rTMS treatment in our present study. However, the mechanisms underlying how the rTMS treatment may influence DA system and further improve clinical symptoms and cognitive deficits of patients with SCZ warrant further investigation.

The limitations of our current study were shown as follows. First, we had a relatively small sample size and may have led to false negative or positive results. Our findings should be confirmed in a large sample from different ethnic populations. Second, one important limitation of our study was the limited 8-week treatment period and no follow-up, which may not be long enough to assess changes in multiple domains of cognition. Therefore, a follow-up study to investigate the efficacy of rTMS for clinical symptoms and cognitive impairments of SCZ is needed to explore their relationship. Third, the subjects in our study were chronically hospitalized older patients, with a longer illness duration and more severe psychopathology than first episode and drug-naive patients with SCZ or typical psychotic outpatients. This limits the generalization of our findings in this study. Fourth, before this study, we only asked the patients verbally whether they had major life stressor or clinical significant emotional disturbance; however, we did not assess these questions by using a rating scale. Fifth, considering that bilateral stimuli reported in the literatures were more likely to improve cognitive functions such as working memory in SCZ, only the left DLPFC was stimulated in the present study, which was a limitation. Sixth, drug use may have an impact on the cognitive function of patients. In this study, however, substance exposure was not assessed by urine analysis or other methods, but only by self-reported drug use.

In summary, the present study indicates that 20 Hz rTMS treatment is beneficial for clinical symptoms and cognitive dysfunction of SCZ. The potential efficacy of 20 Hz rTMS for cognitive dysfunction is important for clinical utilization, since cognitive impairments have been shown to be one of the major obstacles to social dysfunction rehabilitation in patients with SCZ. Thus, rTMS may be a promising cognitive enhancing tool for patients with SCZ as well as other mental disorders. Also, we have found that 20 Hz rTMS is an effective treatment for excited symptoms of SCZ, especially impulse control. Although our findings are encouraging, further investigations are necessary to confirm its efficacy for cognitive deficits in a large sample size of first episode and drug-naïve schizophrenia patients in different ethnic populations with a long follow-up period using a longitudinal design.
